# Active site-specific quantum tunneling of hACE2 receptor to assess its complexing poses with selective bioactive compounds in co-suppressing SARS-CoV-2 influx and subsequent cardiac injury

**DOI:** 10.5455/javar.2021.h544

**Published:** 2021-09-29

**Authors:** Tanzina Sharmin Nipun, Tanzila Ismail Ema, Md. Abdur Rashid Mia, Md. Saddam Hossen, Farzana Alam Arshe, Shahlaa Zernaz Ahmed, Afsana Masud, Fatiha Faheem Taheya, Arysha Alif Khan, Fauzia Haque, Salauddin Al Azad, Md. Al Hasibuzzaman, Mohammad Tanbir, Samin Anis, Sharmin Akter, Sabrina Jahan Mily, Dipta Dey

**Affiliations:** 1 Department of Pharmaceutical Chemistry, Faculty of Pharmacy, International Islamic University Malaysia, Kuantan, Malaysia; 2 Department of Biochemistry and Microbiology, North South University, Dhaka, Bangladesh; 3 Department of Pharmaceutical Technology, Faculty of Pharmacy, International Islamic University Malaysia, Kuantan, Malaysia; 4 Microbiology Major, Faculty of Life Sciences and Medicine, Zhejiang Sci-Tech University, Hangzhou, PR China; 5 Fermentation Engineering Major, School of Biotechnology, Jiangnan University, Wuxi, PR China; 6 School of Medicine, Ningbo University, Ningbo City, PR China; 7 Chattogram Maa-O-Shishu Hospital Medical College, University of Chittagong, Chattogram, Bangladesh; 8 Department of Genetics and Plant Breeding, Bangabandhu Sheikh Mujibur Rahman Agricultural University, Gazipur, Bangladesh; 9 Ministry of Health, People’s Republic of Bangladesh, Dhaka, Bangladesh; 10 Department of Biochemistry and Molecular Biology, Bangabandhu Sheikh Mujibur Rahman Science and Technology University, Gopalgonj, Bangladesh

**Keywords:** SARS-CoV-2, quantum tunneling, hACE2 receptor, supramolecular docking, molecular dynamic, simulation, drug design, cardiac injury

## Abstract

**Objective:**

This research aims to study the target specificity of selective bioactive compounds in complexing with the human angiotensin-converting enzyme (hACE2) receptor to impede the severe acute respiratory syndrome coronavirus 2 influx mechanism resulting in cardiac injury and depending on the receptor’s active site properties and quantum tunneling.

**Materials and Methods:**

A library of 120 phytochemical ligands was prepared, from which 5 were selected considering their absorption, distribution, metabolism, and excretion (ADMET) and quantitative structure–activity relationship (QSAR) profiles. The protein active sites and belonging quantum tunnels were defined to conduct supramolecular docking of the aforementioned ligands. The hydrogen bond formation and hydrophobic interactions between the ligand–receptor complexes were studied following the molecular docking steps. A comprehensive molecular dynamic simulation (MDS) was conducted for each of the ligand–receptor complexes to figure out the values – root mean square deviation (RMSD) (Å), root mean square fluctuation (RMSF) (Å), H-bonds, Cα, solvent accessible surface area (SASA) (Å^2^), molecular surface area (MolSA) (Å^2^), Rg (nm), and polar surface area (PSA) (Å). Finally, computational programming and algorithms were used to interpret the dynamic simulation outputs into their graphical quantitative forms.

**Results:**

ADMET and QSAR profiles revealed that the most active candidates from the library to be used were apigenin, isovitexin, piperolactam A, and quercetin as test ligands, whereas serpentine as the control. Based on the binding affinities of supramolecular docking and the parameters of molecular dynamic simulation, the strength of the test ligands can be classified as isovitexin > quercetin > piperolactam A > apigenin when complexed with the hACE2 receptor. Surprisingly, serpentine showed lower affinity (−8.6 kcal/mol) than that of isovitexin (−9.9 kcal/mol) and quercetin (−8.9 kcal/mol). The MDS analysis revealed all ligands except isovitexin having a value lower than 2.5 Ǻ. All the test ligands exhibited acceptable fluctuation ranges of RMSD (Å), RMSF (Å), H-bonds, Cα, SASA (Å^2^), MolSA (Å^2^), Rg (nm), and PSA (Å) values.

**Conclusion:**

Considering each of the parameters of molecular optimization, docking, and dynamic simulation interventions, all of the test ligands can be suggested as potential targeted drugs in blocking the hACE2 receptor.

## Introduction

The global severe acute respiratory syndrome coronavirus 2 (SARS-CoV-2) pandemic has been deteriorating over time considering the frequencies of spreading and the fatality ratio. Like specific disease-causing microbes, the genetic makeup of SARS-CoV-2 also keeps changing over time, and seven spontaneous mutations have already been reported [1]. There are four types of coronaviruses which belong to the Coronaviridae family, including α-CoV, α-CoV, δ-CoV, and γ-CoV [2]. Coronavirus is a zoonotic virus which encodes many open reading frames because of a positive, single-stranded RNA genome [3]. Although SARS-COV-2 belongs to the *Betacoronavirus* genus, it is more dangerous than the others of the same genus [4]. According to the most recently published data, the mortality rate is higher among aged people, especially over 60 years. Coronavirus disease 2019 (COVID-19) triggers various acute respiratory distress and respiratory failure, leading to cardiac injury, heart failure, and dysfunction in multiple organs [5]. SARS-CoV-2 possesses a total of five genes in its genome, such as the ORF1ab gene that encodes 16 non-structural proteins, envelope (E) gene that encodes envelope protein, spike (S) gene that encodes spike protein, membrane gene that encodes membrane (M) protein, and nucleocapsid gene that encodes nucleocapsid protein [6]. The “spike” proteins exist in two unique conformations, namely pre-fusion and post-fusion. Activation of the S protein is required to transform into its post-fusion confirmation from the pre-fusion status, which leads to membrane fusion which guides coronavirus entry into host cells. S1 and S2 are the two subunits of the spike protein, where the S1 subunit adheres to the cell surface receptor through its receptor-binding domain (RBD). Then the virus fuses with the host membrane through the S2 subunit. Diversified host receptor recognizing capabilities of the S1 subunit is responsible for the multiple variants of SARS-CoV-2. The “S protein– human angiotensin-converting enzyme (hACE2) receptor” complex formation is the main precursor of viral entry and propagation inside the host cells [7]. Individuals with strong immunity show very mild symptoms, whereas people with hypertension, heart and autoimmune diseases, respiratory tract illness, multiple organ damage such as liver, kidney, gastrointestinal tract, and central nervous system become very susceptible to SARS-Cov-2 [8]. Following SARS-CoV-2 infection, several physiological effects become more transparent and acute, like nausea, fever, coughing, vomiting with abdominal pain, and so on [9,10]. 

One of the potent inhibitors of the renin–angiotensin system (RAS) is ACE2, which maintains the equilibrium of blood pressure and fluid balance [11]. The ACE2 receptors are responsible for ceasing the detrimental effects caused by angiotensin II (Ang II), such as vasoconstriction, inflammation, and fibrosis, through degradation of Ang II. After degradation of Ang II, it is converted into Ang I. Ang I acts as a vasodilator and anti-proliferator [12]. ACE2 opposes the actions of Ang II either by indirectly reducing the Ang II synthesis in tissues through cleavage of Ang I or direct hydrolyzing Ang II [13]. Also, their expression varies according to age, sex, and underlying diseases like diabetes, hypertension, and cardiovascular and pulmonary disease [14]. Trimers of the spike protein present in the host receptor ACE2 arbitrates the attachment of SARS-CoV-2 to the cell membrane, which assists the virus’ entry into cells [15]. The virus’ entry through the receptor in the upper respiratory system and lungs significantly damages the cardiac system. It indicates that this virus plays a pathological role in myocardial ACE2 expression [16]. Studies have shown that SARS-CoV-2 has more affinity toward ACE2 receptors, along with other reports depicting those organs are receptive to SARS-CoV-2 infection, demonstrating higher levels of ACE2 expression [17]. Attachment of the spike protein with the ACE2 receptor leads to a decrease in ACE2, which results in an upregulation of Angiotensin II, causing an imbalance in RAS, which is unfavorable for the patients. This dysregulation implicates the onset and pathogenesis of hypertension [18]. Hence, the presence of the host ACE-2 receptor is the gateway for the SARS-CoV-2 virus’ entry into host cells which has been highlighted in many studies recently [1]. Thus, variations present in spike protein and host ACE-2 receptor binding sites can considerably decrease the virus’ effects on the patient’s physiology [19]. Ang1–7 has many diverse roles, such as anti-inflammation, antioxidant, vasodilatory, and natriuretic effects regulated by the G-protein-coupled receptor [20], which can be misregulated S–hACE2’ complexing creating heart injury.

Considering all of the factors above, the current study aims to identify the interactions and target specificity of selective biosynthetic ligands to the hACE2 receptor, depending on its active site detection and quantum tunnel profiles. The hydrogen bindings and hydrophobic interactions responsible for blocking the S–hACE2 receptor complex formation were also analyzed. Besides, to figure out the significance of molecular dynamic simulation in evaluating the efficacies of the test bioactive components was also conducted to impede SARS-CoV-2 influx mechanism and subsequent heart injuries, sophisticated computer programming, and biostatistical algorithms were used.

## Material and Methods

### Construction of the library of phytochemical compounds

A library of phytochemical aromatic compounds was prepared with 120 ligands, followed by a thorough literature review. Ligand screening has been conducted through comprehensive profiling of their physiochemical properties based on PubChem (https://pubchem.ncbi.nlm.nih.gov/), a National Center for Biotechnological Information affiliated directory of chemical substances and biological assays.

### Screening the ligand library via ADMET and QSAR profiling for ligand validation 

PubChem (https://pubchem.ncbi.nlm.nih.gov/) has been utilized for collecting all the 3D structures of the targeted ligands in the form of structure data file (SDF). To study their pharmacokinetic features, such as absorption, distribution, metabolism, excretion, and toxicity, all the 120 ligands have been checked via the absorption, distribution, metabolism, and excretion (ADMET) profiling that interprets the ligand’s propensity inside the body [21]. For generating the ADMET profile of the selected test ligands, “Swiss ADME” (http://www.swissadme.ch/index.php) and “Molinspiration Cheminformatics” (https://www.molinspiration.com/cgi-bin/properties) was employed. For secondary identification of ADMET, “pkCSM” (http://biosig.unimelb.edu.au/pkcsm/prediction) was implemented where the ligands’ toxicity parameters were emphasized. Subsequently, the potential ligands were run via “admetSAR 2” (http://lmmd.ecust.edu.cn/admetsar2/) for determining the quantitative structure–activity relationship (QSAR). After considering the ADMET and QSAR profiling, six ligands that showed promising values and properties in terms of their ADMET and QSAR were selected for supramolecular docking and molecular dynamic simulation against the control ligand serpentine. All the ligands were optimized using University of California San Francisco (UCSF) Chimera (version 1.14) [22]. The Gasteiger approach was applied to minimize energy by lowering the accumulative charge on ligands to zero [23]. The optimized ligands were then converted into a “mol2 file” for conducting molecular docking.

### Preparation of the macromolecule 

The 3D crystal structure of human angiotensin-converting enzyme-related carboxypeptidase (hACE2) was gathered from the database of protein data bank (PDB ID: 1R4L and Resolution 3.00 Å). The protein’s crystal structure was optimized using UCSF Chimera (version 1.14) to obtain proper orientation, size, and rotations [23–25]. The non-standard amino acid, ions, water molecules, and ligands were deleted from the protein crystal structure during optimization to avoid interacting with the undesired parts of the receptor. In addition, to assure congenial performance during docking, missing hydrogen atoms were added to the macromolecule [26]. The minimized energy was calculated by YASARA (https://www.yasara.org) [27].

### Active site prediction of the receptor macromolecule 

The supramolecular docking pose of the optimized hACE2, indicating the best active site, was determined and validated using the COACH-D (https://yanglab.nankai.edu.cn/COACH-D/) algorithm [28]. Using the algorithm, a total of three suggestive binding poses were found, among which the best one was selected, considering the number of amino acid residues involved and the projected binding affinity (kcal/mol). Finally, the best active site predicted file was subjected to quantum tunneling. 

### Quantum tunneling on the best active site of the receptor

To gain a better understanding of the intended modalities of ligand accommodation within the hACE2 receptor, various protein tunnels were found using the Schrodinger algorithm and the CAVER Web 1.0 tools (https://loschmidt.chemi.muni.cz/caverweb/). A total of 24 tunnels were found from the initial prediction. Finally, considering the tunnel length and radius (Å), the six most viable tunnels were determined using CAVER 3.0 [29]. The tunnels were studied to validate the pre-identified best active site of the receptor for proceeding a successful supramolecular docking because protein tunneling explains efficient electron transport via protein junctions which is a precursor of super-docking [30]. 

### Point-specific molecular docking

Molecular docking of the selected optimized ligands was undertaken using PyRx version 0.8 to scrutinize the binding efficacy of the optimized protein–ligand complexes [31]. The desired ligands and macromolecule were transformed to the format “pdbqt during the molecular docking operation. The root mean square deviation (RMSD) (Å) and binding affinity (Kcal/mol) output files were saved as comma-separated values files.

### Post-docking analysis

For the initial visualization and qualitative receptor–ligand interactions analysis, Discovery Studio Visualizer (version 3.0) and PyMOL (version 2.4.1) were used sequentially, and the output files were saved as PDB files. Afterward, the quantitative hydrophobic interactions and the number of hydrogen bond formations between each of the protein–ligand complexes were analyzed using LigPlot+ (version 2.2) [32] before conducting the molecular dynamic simulation. 

### Molecular dynamics simulation

In the beginning, the ligand-free macromolecule 1R4L was subjected to dynamic simulation for 10 ns to investigate its natural physical alteration and its reaction with surrounding ions and water molecules utilizing the web-based dynamic simulator CABS-flex 2.0 web-based simulation (http://biocomp.chem.uw.edu.pl/CABSflex2/) [33]. Subsequently, the protein–ligand complexes were preliminarily subjected to the ligand and receptor molecular dynamics (LARMD) simulation system (http://chemyang.ccnu.edu.cn/ccb/server/LARMD/index.php) [34]. LARMD was run for 3.1 ns to understand the outcomes of each protein–ligand complex independently in terms of solvent accessible surface area (SASA), Debye–Waller factor for thermostability (B-factor), root mean square fluctuation (RMSF), principal component analysis, and RMSD analysis [32]. Finally, molecular dynamic simulation of the ligand–receptor complexes was conducted up to 20 ns using Desmond (Desmond, Schrödinger, LLC, NY) software package to investigate RMSD (Å), RMSF (Å), Rg (nm), H-bonds, SASA (Å^2^), molecular surface area (MolSA) (Å^2^), and polar surface area (PSA) (Å) of each protein–ligand complex [35]. The box dimension was fixed at X:Y:Z, and nullifying ions (Na+) were added as needed to get the desired results. The probe radius was adjusted to 1.4 Å to investigate the molecular surface area and solvent-accessible surface area.

### Statistical analysis and graphical representation

The protein–ligand complexes have been improved and visualized using the software packages listed above. In addition, data mining and statistical interpretation of the values of RMSD (Å), RMSF (Å), Rg (nm), H-bonds, SASA (Å^2^), MolSA (Å^2^), and PSA (Å) from the molecular docking and molecular dynamics simulation were accomplished by GraphPad Prism version 8.0.1 software package (for Mac OS) [36,37] and R programming (version R-4.0.2 for Linux) [38,39].

**Figure 1.**
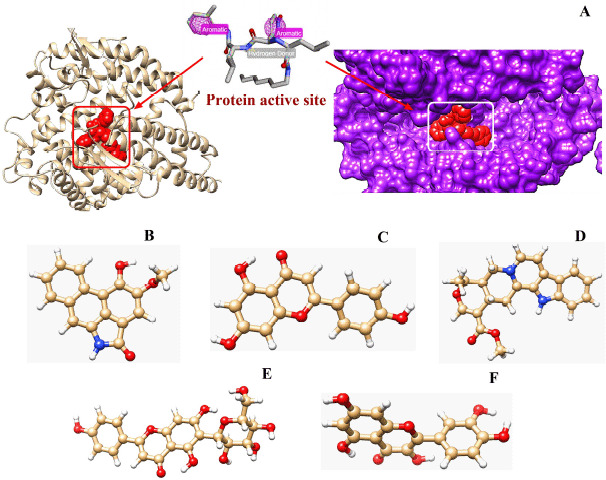
Illustration of all the optimized ligands and receptor macromolecule simultaneously. The hACE2 receptor (PDB ID: 1R4L) with its active site for complexing with the ligands (A); the drone-like structure refers to the main conformation of the pocket (A). Among the ligands serpentine (D) was the control and the others are the test ligands means – piperolactam A (B), apigenin (C), isovitexin (E), and quercetin (F).

## Results

### Protein active site detection and quantum tunneling

**Figure 2.**
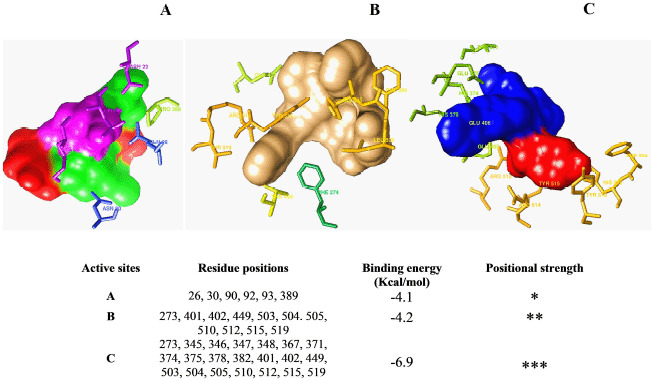
Selection of different active sites of the hACE2 receptor protein considering the binding energy (Kcal/mol) and the number of the amino acids involved at the docking region.

**Figure 3.**
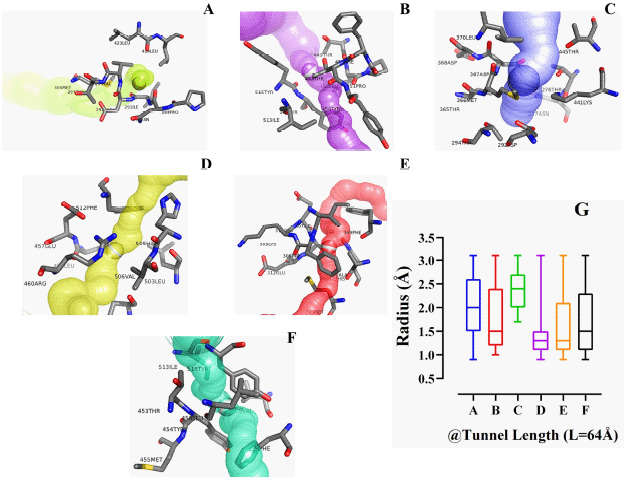
Illustration of the protein tunnels along with the tunnel length (Å) and radius (Å) of each of the tunnels for allocating any ligand in the super docking position.

The energy level of the hACE2 receptor protein was minimized from −52407.7 to −113291.2 KJ/mol, as a result of the optimization process (Fig. 1A). The three protein active sites showed their projected binding affinities as −4.1kcal/mol (Fig. 2A), −4.2Kcal/mol (Fig. 2B), and −6.9Kcal/mol (Fig. 2C). Considering the number of amino acid residues and binding energy, the third pose of the protein active site (Fig. 2C) was taken for quantum tunneling. For the third active site position, six tunnels were identified (each at 64 Å length) with the radius (Å) ranged between 0.83Å and 3.17Å (Fig. 3). The tunnels are the suggested routes for molecular super docking. 

### Pharmacokinetic profiles of the ligands

In this study, five ligands, including control, were picked from a library of 120 renowned pharmacophores following their QSAR and ADMET profile analysis, which are piperolactam A (Fig. 1B), apigenin (Fig. 1C), isovitexin (Fig. 1E), and quercetin (Fig. 1F) as test ligands, whereas serpentine (Fig. 1D) was the control (Table 1). Based on the physicochemical analysis, there were no violations of Lipinski’s rules in any of the tested ligands. While observing the pharmacokinetics analysis, only piperolactam A among the ligands showed partial AMES toxicity. In addition to an excellent excretion rate, these five ligands had maximum tolerated dosages ranging from −0.219 to 0.649 log mg/kg/day. The intestinal absorption of serpentine (control), apigenin, and piperolactam A were typically > 90%, whereas quercetin and isovitexin were 77.207% and 64.729% respectively. The blood–brain barrier range of the ligands, namely apigenin, isovitexin, piperolactam A, and quercetin, were −0.734, −1.375, −0.397, and −1.098, respectively, but control serpentine was 0.257. Except for serpentine, all ligands demonstrated hepatotoxicity and their LD50 ranged between 2.471 and 3.675 (Table 1).

### Supramolecular docking

Supramolecular docking showed that isovitexin exhibited the highest binding affinity (−9.9 Kcal/mol) toward the enzyme, which was also higher than the control ligand, serpentine (−8.6 Kcal/mol). Besides isovitexin, quercetin also showed a higher binding affinity value (−8.9 Kcal/mol) than the control ligand. On the other hand, apigenin demonstrated a lower binding affinity value (−8.1 Kcal/mol) than serpentine. Furthermore, similar binding affinity values were found in both piperolactam A-1R4L and serpentine-1R4L complexes (Table 2). Table 2 also depicts the RMSD values of five ligands, including the control ligand, along with binding affinity. It was determined from the docking results that the other four ligands, namely quercetin, isovitexin, piperolactam A, and apigenin, showed lower values of RMSD (6.186, 8.951, 4.847, and 17.556 Å, respectively) compared to the control ligand (RMSD/UB 20.224 Å). The lowest RMSD values were exhibited among the five ligands by piperolactam A (RMSD/UB 4.847 Å and RMSD/LB 2.351 Å), whereas the control ligand showed the highest values of RMSD (Table 2). 

**Table 1.** Complete QSAR and ADME/T physiochemical and pharmacokinetic profiling of selected ligands.

Name of ligand and ID	Physiochemical properties								Pharmacokinetic criteria					
	MoW	LogP	H-Ac	H-Do	NRB	BBB	NVL	DL	IA	TC	AT	LD50	HT	MTD
Serpentine (control) CID 73073	348.402	3.4052	5	0	1	0.257	0	yes	97.616	0.931	No	3.675	yes	−0.219
Apigenin CID 5280443	270.240	2.5768	5	3	1	−0.734	0	yes	93.5	0.566	No	2.450	no	0.328
Isovitexin CID 162350	432.381	0.0917	10	7	3	−1.375	1	no	64.729	0.442	No	2.558	no	0.649
Piperolactam A CID 3081016	265.268	3.2729	3	2	1	−0.397	0	yes	95.084	0.046	Yes	2.634	no	−0.128
Quercetin CID 5280343	302.238	1.9880	7	5	1	−1.098	0	Yes	77.207	0.407	No	2.471	no	0.499

QSAR, Quantitative structure–activity relationship; ADME/T, Absorption, distribution, metabolism, excretion, and toxicity; MoW, molecular weight, g/mol; LogP, Predicted octanol/water partition coefficient; H-Ac, No. of hydrogen bond acceptor; H-Do, No. of hydrogen bond donor; NRB, No. of rotatable bonds; BBB, Blood–Brain Barrier; NLV, No. of Lipinski’s rule violations; DL, Drug likeness; IA, Intestinal absorption, % absorbed; TC, Total clearance, log ml/min/kg; AT, AMES toxicity; LD50, Oral rat acute toxicity; HT, Hepatotoxicity; MTD, Maximum tolerated dose for human, log mg/kg/day.

NRB and NVL were taken from SwissADME and the rests from pkCSM.

**Table 2.** Pharmacokinetics profiling of ADMET and QSAR for ligand validation.

Macromolecule	Ligand	Binding affinity (Kcal/mol)	RMSD (Å)	
			Upper Bound (Å)	Lower Bound (Å)
**1R4L**	Serpentine (control)	-8.6	20.224	18.545
**1R4L**	Quercetin	-8.9	6.186	3.72
**1R4L**	Isovitexin	-9.9	8.951	5.004
**1R4L**	Piperolactam A	-8.6	4.847	2.351
**1R4L**	Apigenin	-8.1	17.556	17.187

RMSD, Root mean square deviation; UB, Upper bound; LB, Lower bound;

1R4L was taken as standard macromolecules for hydrolyzing lignin and cellulose, respectively; PubChem CID: serpentine (73391), quercetin (5280343), isovitexin (162350), piperolactam A (3081016), apigenin (5280443).

**Figure 4.**
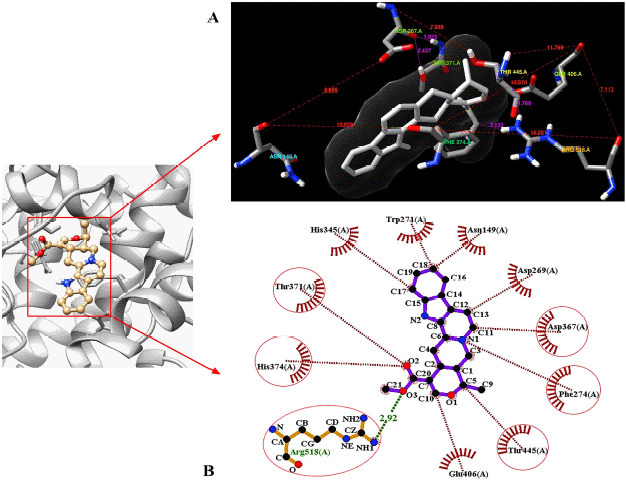
Identification of the super docking position of the control ligand (serpentine) inside the hACE2 receptor as well as the hydrogen and hydrophobic interactions involvement inside the ‘serpentine–hACE2 receptor’ complex. The distances among the amino acid residues are mentioned using red lines where the hydrogen bonds are the pink lines (A) as 3D confirmation. Besides, the most stable hydrogen bonds (green line) and hydrophobic interactions (red lines) are represented in 2D.

**Figure 5.**
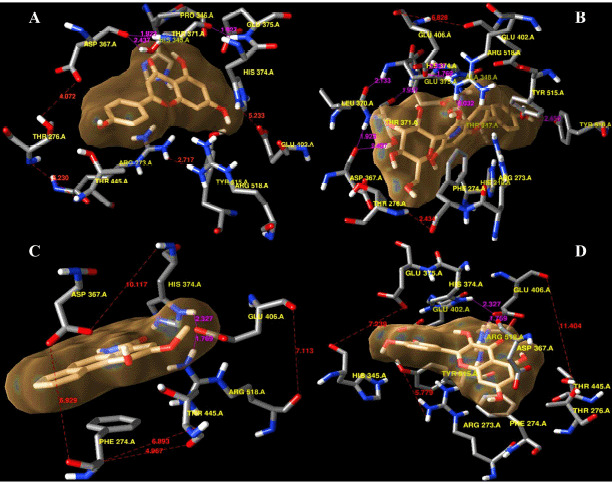
The super docking positions of the test ligands are portraited when complexed with the hACE2 macromolecule. The formation of hydrogen bonds and hydrophobic interactions are mentioned using pink and red lines, respectively. The complexes are apigenin–1R4L (A), isovitexin–1R4L (B), piperolactam A–1R4L, (C), and quercetin–1R4L (D).

### Post-molecular super docking analysis 

Serpentine, the control ligand, showed only one hydrogen bond, namely Arg518 (2.92 Å), alongside the five hydrophobic bond residues, such as Thr445, Phe274, Asp367, His374, and Thr371, interacting with the macromolecule 1R4L (Fig. 4). The interaction of apigenin with 1R4L, four hydrogen bond residues, namely Glu402 (2.87 Å), His374 (3.08 Å), Pro346 (2.73 Å), and Thr445 (2.57 Å), have been detected (Fig. 5A). In the profiling of the apigenin–1R4L complex four hydrophobic residues, namely Glu375, Thr371, Phe274, and Tyr515, have been observed (Table 3). Isovitexin developed a compacted interaction with its ligand via four hydrogen bond residues, including Thr371 (3.25 Å), Asp367 (2.82 Å), Glu406 (3.12 Å), and Arg (2.82 Å), along with hydrophobic interactions such as Phe274, Glu375, Pro346, His345, Thr347, His374, Tyr515, and Glu402 (Fig. 5B). In the case of piperolactam A, the hydrophobic residues residing inside are Arg518 (3.02 Å) and Thr (3.10 Å) only, whereas the involved hydrophobic residues are Glu406, Phe274, and Thr276 (Fig. 5C). Finally, the quercetin–1R4L complex displayed Arg518 (2.89 Å), Glu406 (2.97 Å), and Pro (2.87 Å) as three hydrogen bond interactions (Fig. 5D) and Asp367, Phe274, Thr371, Glu375, and His374 as five hydrophobic bond residues (Table 3).

**Figure 6.**
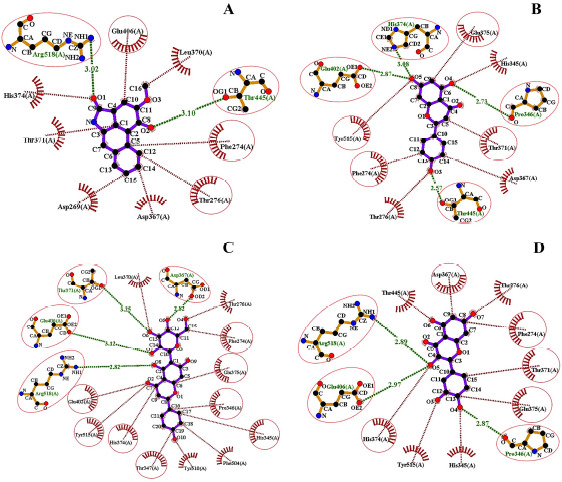
Identification of the ligand–receptor stabilities following the number of hydrogen bonds (green lines) and the noncovalent interactions (red lines) among the ligand and the amino acid residues. hACE2 receptor protein is complexed with piperolactam A (A) apigenin (B), isovitexin (C), quercetin (D).

Following the hydrophobic bond interactions shown in all ligand–receptor complexes, isovitexin confirmed the nearest contiguity comprising eight amino acids (Fig. 6C), whereas piperolactam A showed only three (Fig. 6A). Interestingly, the other ligands displayed four or five hydrophobic bond interactions. Moreover, isovitexin also holds the highest number of hydrogen bond interacting residues compared to other ligands and the control ligand. There is a total number of four hydrogen bond interacted residues in isovitexin. Even though apigenin possesses the same number of hydrogen bond residues as isovitexin (Fig. 6B), the greatest number of hydrophobic bond interacting residues are observed in isovitexin. Quercetin possesses three hydrogen bonds, but the amino acid residues are less involved in it (Fig. 6D).

**Table 3.** Analysis of the binding affinities of the candidate ligands with the hACE2 receptor

Macromolecule	Ligand	Amino acid involved interactions	
		Hydrogen bond interactions	Hydrophobic bond interactions
**1R4L**	Serpentine	Arg518(2.92 Å)	Thr445, Phe274, Asp367, His374, Thr371
**1R4L** (Fig. 1D)	Quercetin	Arg518(2.89 Å), Glu406(2.97 Å), Pro (2.87 Å)	Asp367, Phe274, Thr371, Glu375, His374
**1R4L** (Fig. 1C)	Isovitexin	Thr371(3.25 Å), Asp367(2.82 Å), Glu406(3.12 Å), Arg (2.82 Å)	Phe274, Glu375, Pro346, His345, Thr347, His374, Tyr515, Glu402
**1R4L **(Fig. 1A)	Piperolactam A	Arg518(3.02 Å), Thr(3.10 Å)	Glu406, Phe274, Thr276
**1R4L** (Fig. 1B)	Apigenin	Glu402(2.87 Å), His374(3.08 Å), Pro346(2.73 Å), Thr445(2.57 Å)	Glu375, Thr371, Phe274, Tyr515

### Molecular dynamic simulation (20 ns) 

**Figure 7.**
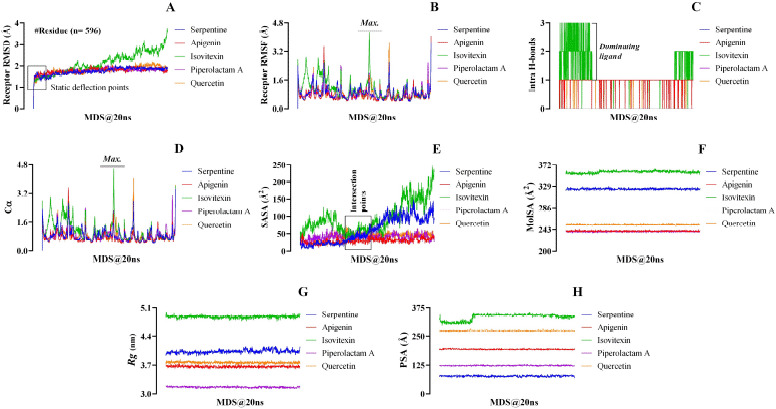
Molecular dynamic simulation (20 ns runtime) of the ligand–receptor complexes’ results, means – RMSD (A), RMSF (B), H-bonds (C), Cα (D), SASA (E), MolSA (F), Rg (G), and PSA (H).

In total, eight parameters have been considered to check the fluctuation profiles of the docked complex from the molecular dynamic simulation (Fig. 7). During 20 ns of the simulation period, 596 interactive amino acid residues of 1R4L were harvested among 1001 different frames.

From the molecular dynamic simulation analysis, the control ligand serpentine was found to have a range of RMSD values within 0 Ǻ–2.07 Å. Isovitexin displayed the highest RMSD value of 3.752Ǻ in comparison to all test and the control ligands. On the contrary, piperolactam A showed the lowest RMSD value at 2.057 Å. However, quercetin showed higher and apigenin exhibited lower RMSD values at 2.196 Å and 2.062 Å, respectively, than the control serpentine (Fig. 7A). Similarly, isovitexin showed a significantly higher RMSF fluctuation range from 0.520 Å to 4.268 Å, as compared to the control ligand (0.424 Å–3.704 Å) and the rest of the three experimental ligands (Fig. 7B). Besides, apigenin (0.417 Å–4.054 Å) and quercetin (0.427 Å–3.944 Å) also exhibited higher RMSF fluctuations compared to serpentine. On the other hand, no significant difference in fluctuations was observed between control and piperolactam A (0.428 Å–3.883 Å).

An intramolecular hydrogen bond was absent in the docked complexes containing the control ligand, serpentine, and piperolactam A (Fig. 7C). On the contrary, quercetin and apigenin possessed only one intramolecular hydrogen bond. The isovitexin–1R4L complex exhibited the highest number of intramolecular hydrogen bond interactions (Fig. 7C). 

For the control ligand serpentine, the range of interactive alpha carbon atoms (Cα) was observed between 0.402 and 3.443, which was found to be very close to the apigenin (0.396–3.517) and piperolactam A (0.412–3.579). On the other hand, a higher range of interactive alpha carbon atoms was observed in both isovitexin–1R4L (0.502–4.573) and quercetin–1R4L (0.416–4.056) complexes, compared to the serpentine–1R4L complex. However, isovitexin showed the highest interactive alpha carbon atoms range during the simulation period with the protein (1R4L) compared to all other ligands (Fig. 7D). 

In this study, SASA revealed the level of exposure of 597 amino acid residues of the enzyme 1R4L, associated with ligand to solvent (water) and pharmacokinetic strength of lead molecules, including quercetin, isovitexin, piperolactam A, and apigenin along with the control ligand, serpentine. In all cases, the water probe radius of 1.4 Å and the ratio of the total area to energy for polar region 9598.23 and apolar region 14050.71 were observed with no gradient of calculation and no unknown area (Table 4). The ligand isovitexin showed the highest range of a fluctuation profile in terms of SASA (24.798 Å^2^ –245.328 Å^2^) compared to all test ligands, including the control. On the contrary, apigenin (8.955 Å^2^–53.109 Å^2^), piperolactam A (11.134 Å^2^–79.483 Å^2^), and quercetin (12.919 Å^2^–71.398 Å^2^) exhibited a lower range of SASA fluctuation profile compared to the serpentine (5.281 Å^2^–149.644 Å^2^) (Fig. 7E).

To determine the MolSA, a probe radius of 1.4 Å (equivalent to the van der Waals surface area of a water molecule) was used. Throughout the 20 ns of the simulation period, the profile of MolSA values fluctuated in different ranges. When compared to all test ligands, including the control (Fig. 7F), isovitexin demonstrated the highest MolSA value (364.806 Å^2^), whereas piperolactam A exhibited the lowest (242.419 Å^2^). However, quercetin and apigenin displayed lower molecular surface area values of 256.595 Å^2^ and 244.140 Å^2^, respectively, than that of serpentine (329.282 Å^2^). 

**Table 4.** Solvent accessible surface area (Å2) referring the area to energy ratio over the entire dynamic simulation process (100 ns) with polar and apolar regions precisely

Macromolecule	Ligand	Water Probe Radius ( Å )	Gradient in Calculation	Total number of Residue	Total area/Energy		
					Polar	Apolar	Unknown
1R4L	Serpentine	1.400	No	597	9598.23	14050.71	0.00
1R4L	Quercetin	1.400	No	597	9598.23	14050.71	0.00
1R4L	Isovitexin	1.400	No	597	9598.23	14050.71	0.00
1R4L	Piperolactam A	1.400	No	597	9598.23	14050.71	0.00
1R4L	Apigenin	1.400	No	597	9598.23	14050.71	0.00

WPR, Water probe radius; GIC, Gradient in calculation; TNR, Total no. of residues.

In the molecular dynamic simulation course, the highest fluctuation of the radius of gyration (Rg) value was observed in the docked complex containing isovitexin, varying in the range between 4.760 and 4.992 nm. On the contrary, the ligand piperolactam A exhibited the lowest score between 3.111 and 3.211 nm. The control ligand, serpentine, displayed Rg values ranging from 3.913 to 4.164 nm. Besides, quercetin (3.696–3.849 nm) and apigenin (3.597–3.743nm) generated Rg values of a lower range than that of the control ligand (Fig. 7G).

Although all ligands exhibited a higher range of polar surface area than the control, isovitexin showed the widest range (297.167 Å–356.201 Å) compared to all the test and control ligands. The PSA values found for serpentine ranging from 67.614 Å up to 86.226 Å. Notable fluctuations were also observed among quercetin, piperolactam A, and apigenin with the PSA values of 284.805 Å, 129.34 Å, and 200.676 Å, respectively (Fig. 7H). 

## Discussion

The World Health Organization identified the Delta variant [Phylogenetic Assignment of Named Global Outbreak lineage designation B.1.617.2], initially found in India, as a variant of concern in May 2021, and linked it to an epidemic resurgence in the United Kingdom. The lineage comprised three subtypes (B1.617.1, B.1.617.2, and B.1.617.3), each with a different set of “Spike mutations” in the N-terminal domain (NTD) and the RBD that might help them evade the immune system. “B.1.617.2,” commonly known as variation Delta, is thought to spread more quickly than other variants [40].

From the understanding of the pathophysiology of the SARS-CoV-2, it can be said that the spike protein acts as a ligand by binding with the hACE2 receptor facilitating the viral particle entry into the host cell. Thus, through modulator proteins as ligands, it can be possible to allosterically regulate the spike protein’s binding activity [41]. Phytochemicals are naturally synthesized in plants and are well known to have numerous therapeutic properties, having several studies reported on its competence against diseases, additionally offering a variety in compounds chosen [42]. The binding of these phytochemicals with host protein ACE2 as a non-competitive molecule can confer antiviral efficacy by disrupting spike protein binding to the hACE2 receptor [43]. For the current *in silico* study, the chosen pharmacophore compounds against the ACE2 receptor are quercetin, isovitexin, piperolactam A, and apigenin, keeping SERPENTINE as the control ligand (Fig. 1B–F). 

### ADMET and QSAR

From the assessment of the physicochemical properties, only isovitexin among the five ligands was observed to partially violate the Lipinski rule of 5, leading to its deflection than the other ligands exhibited drug-likeness properties [25]. All ligands displayed prominent excretion rates along with acceptable ranges of maximum tolerated dosages. More than 90% intestinal absorption was witnessed for the ligands serpentine (control), apigenin, and piperolactam A, whereas lower than 90% was displayed by quercetin and isovitexin. AMES toxicity was tested positive for piperolactam A only. The blood–brain barrier range of the ligands apigenin, isovitexin, piperolactam A, and quercetin came out as negative, whereas the value for serpentine only came out as positive. With the control ligand being the exception, the hepatotoxicity came negative for the rest of the four ligands (Table 1). In the case of the LD50 dosage, serpentine acted abnormally again, whereas the LD50 range for the other four ligands were acceptable ranges. 

### Protein active site detection and quantum tunneling 

A web server named “CASTp” (http://sts.bioe.uic.edu/castp/index.html?3trg) was used for the prediction of the active binding site of the ACE2 receptor, but it was able to give only a qualitative-predicted result (Fig. 1A). COACH-D algorithm was utilized for the quantitative assessment of the predicted active site of the hACE2 receptor protein [28,34], which generated quantitative data of a total of eight best calculating binding poses. Upon analyzing the binding energy and amino acid residue positions, the top three binding poses were chosen (Fig. 2) out of the eight resulted ones. For the authentication of the data and running a perfect supramolecular docking, the third binding pose was further subjected to Caver 3, to carry out quantum tunneling of the best active site binding pose [44]. In total, 24 quantum tunnels were developed by the Caver 3 from which the six most viable were chosen from the binding active site of the protein (Fig. 3A–F). The graph (Fig. 3G) depicts the quantitative analysis where the radius and tunnel length are dependent and independent variables, respectively, the radius aid in deducing the ligand’s length, width, and height [45]. The tunnel was observed to be surrounded by amino acid residues, while the ligand paved its way through the tunnel (Fig. 3A). This very tunnel has been demonstrated in a graph with a radius of 2.0 Ǻ. The same phenomena were witnessed in Figure 3B and C, but with different radii of 1.5 Ǻ and 2.4 Ǻ, respectively [46]. Figure 3D shows a similar frame to 3B, where the ligand is in the tunnel center exhibiting a radius of 1.25 Å. Figure 3E shows a slightly different picture where the scattered amino acids are far away from the ligand. However, the radius’ value of Figure 3E is identical to 3D, which is 1.25 Å. Lastly, in Figure 3F, fewer amino acids around the ligand depicted a radius with a value of 1.5 Å. By studying the quantitative analysis of the graph, it was concluded that Figure 3C tends to have the highest value which is 2.4 Å, and the lowest value is 1.25 Å found in Figure 3E and F.

### Molecular optimization and docking

Molecular docking is a computational modeling technique that visualizes predictive pharmacophore complexes that occur between the ligand and the receptor protein via running them on program PyRx 0.8, a virtual screening tool which is based on Autodock Vina for the comprehension of the binding affinity between the ligand and the macromolecule when it is subjected to any condition depending on binding scores [47]. Before carrying out the molecular docking, UCSF Chimera Software Package (Version 1.14) was used to optimize the protein of interest and the five ligands. This software was also used to visualize the manner in which the binding positions if and when supramolecular docking will take place between the macromolecule and the ligand. Figure 4 shows the simultaneous qualitative (Fig. 4A) and quantitative (Fig. 4AB) forms of the control ligand serpentine complexed with the macromolecule hACE2 receptor. The qualitative form was developed via the UCSF Chimera Software, and here the predictive location of the hydrogen bonds between the specific amino acids are indicated using the magenta line. This was further conducted by the java interface-run program, LigPlot+ V.2.2 tool, to identify and visualize the hydrogen bonds and hydrophobic interactions between the ligand and the peripheral amino acid residues [32]. 

The protein was modeled in supramolecular docking with four ligands using the UCSF Chimera. The post-docking prediction of the binding poses on the ACE2 receptor through which the corresponding ligands bound to its protein, depending on the quantum tunneling mechanism conducted. The intermolecular spaces (red lines) and projected hydrogen bonds (magenta lines) are shown in Fig. 5. 

The PyMOL-generated results (PDB format) were then subjected to visualization in the Ligplot+, which then displayed the number of hydrogen bond interactions and hydrophobic interactions of the ligand with the amino acids residing within its periphery (Fig. 6). Isovitexin (Fig. 6C) and apigenin (Fig. 6B) possessed the highest number of hydrogen bond interactions, while in the case of hydrophobic interactions, isovitexin tops apigenin. On the contrary, piperolactam A displayed the least number of hydrogen bonds along with hydrophobic interactions (Fig. 6A).

It was observed that the isovitexin complexed with the receptor possessed the highest binding affinity (-8.9 kcal/mol) compared to the control ligand serpentine (−8.6 Kcal/mol). This may be because the number of hydrogen bonds of the “isovitexin–1R4L” complex was higher than the “serpentine–1R4L” complex, whereas “apigenin–ACE2” protein had the least binding affinity value but was higher than the docked complex containing control ligand hydrogen bond, which plays a crucial role in protein–ligand binding by stabilizing the docked complex [48]. Besides hydrogen bonds, the higher number of hydrophobic interactions within the docked complex may also increase the binding affinity of the ligand toward the target protein [49,50]. Among the four test and control ligands, isovitexin has a higher number of carbonyl and hydroxyl moieties that may contribute the highest binding affinity of this ligand toward the protein.

### Molecular dynamic simulation (20 ns) 

In this research, Desmond (Desmond, Schrödinger, LLC, NY) has been used for molecular dynamic simulation (MDS) that was operated to investigate the receptor RMSD, receptor RMSF, Intra H-bonds, Cα, SASA, MolSA, Rg, and PSA for 20 ns. The RMSD values are considered to measure the average shift in a set of atoms for a specific frame with a reference frame [51]. This parameter governs a significant role in assisting the comparisons among different molecular structures and narrows down the extensive list of predictive conformations to a smaller set [52]. The greater the RMSD value, the less stable the docked complex during the simulation period, and vice versa [53]. In terms of the lowest RMSD values, all the experimental and control ligands demonstrated equal stability in their docked complexes. However, the stability pattern of the ligands, based on the RMSD values was piperolactam A > apigenin > serpentine > quercetin > isovitexin (Fig. 7A). From this trend, it was observed that the “piperolactam A–1R4L” complex showed the highest stability, whereas the “isovitexin–1R4L” complex exhibited the lowest stability during MDS analysis. The RMSF values were estimated for 20 ns to analyze the effect of a test ligand on 1R4L protein. RMSF value implies the denaturation tendency of a receptor protein at each point of the temporal trajectory (Fig. 7B). The higher the RMSF value, the lower the stability of the protein–ligand complex during the MDS and vice versa [53]. This phenomenon occurs due to a change in protein structure induced by ligand interaction when higher RMSF is attributed to the presence of tightly bonded structures like an alpha helix and beta-strands. At the same time, a lower RMSF refers to lose structures like coils, bends, and turns [54]. The ligand isovitexin exhibited the highest RMSF values than the control serpentine and the other experimental ligands, indicating lower stability due to enhanced flexibility. The control showed the least RMSF values to form the most stable complex. On the other hand, the docked complexes containing quercetin, piperolactam A, and apigenin showed lower stability than the control (Fig. 7B).

The hydrogen bond networks play a crucial role in strengthening the binding affinity of the protein and the ligand [55]. In our study, the “isovitexin–1R4L” complex showed the highest number of intramolecular hydrogen bond interactions among all ligands, including the control, indicating the most stable complex during the 20 ns of the simulation period. On the other hand, quercetin and apigenin exhibited a bit higher stability compared to control. The simulation findings demonstrated that intramolecular hydrogen bonds were absent in the docked complexes containing serpentine and piperolactam A, indicating the least stability in those complexes (Fig. 7C). The alpha-carbon atom is one of the mother parameters of MDS analysis which plays a vital role to get information on the motions of the protein–ligand complexes during simulation periods (Fig. 7D). Fastest (i.e., narrow region of alpha carbon atom) motions indicate the most biologically unstable conformations [56]. Therefore, the findings revealed that narrow regions of Cα were exhibited by the docked complex containing serpentine, apigenin, and piperolactam-A, indicating the fastest motion of their complexes, which resulted in the unstable conformations. However, the “isovitexin–1R4L” complex showed the widest region of carbon alpha, indicating stable conformations. Furthermore, the docked complex containing quercetin also exhibited a broader region of Cα compared to control, indicating the more stable conformation formed by the “quercetin–1R4L” complex than the serpentine–1R4L complex (Fig. 7D). There were no significant differences observed among serpentine, apigenin, and piperolactam A. 

To better understand the effective interaction between the macromolecule and ligand, the SASA values are used to interpret the interactions between the surface of the docked complex and water molecules in which the protein–ligand was submerged. For the stability of the proteins, hydrophobic interactions form between the non-polar amino acids through defending by polymer shielding in hydrophobic, which reduced SASA values [25,57]. The MDS analysis (Fig. 7E) revealed that serpentine (control) exhibited the most stability following the lowest SASA value. On the other hand, quercetin, and piperolactam A followed higher values of SASA, but isovitexin demonstrated the highest SASA value. The stability of a protein is also connected to its MolSA; consequently, a significant change in the MolSA of a complex might result in instability, which is highly undesirable [58,59]. After 20 ns of the simulation period, it was found that isovitexin showed the highest MolSA value among all five ligands (Fig. 7F). Thus, isovitexin can be considered the least stable and unfavorable in comparison to the other ligands. On the contrary, the most stable complex was formed between piperolactam A and 1R4L. Additionally, the docked complexes containing apigenin and quercetin were also displayed higher stability than the serpentine–1R4L complex (Fig. 7F). 

The radius of gyration (Rg) is the benchmark to determine whether a structure has a stable, compact and folded conformation. The higher the Rg value, the more likely the ligand is flexible, thus possessing an unstable conformation. On the contrary, lower Rg values suggest a dense and closely packed structure [60]. Isovitexin exhibited the highest gyration radius, indicating the most flexible and unstable compound compared to the rest of the three experimental and control ligands. On the other hand, quercetin and apigenin exhibited higher stability than serpentine. From the simulation analysis, piperolactam-A was found to be the model ligand in terms of Rg (nm) value (Fig. 7G). 

PSA is a crucial factor along with the lipophilicity to determine the ability of drugs to cross the blood–brain barrier [61]. The normal range of PSA for the substance (*X*) is 40 Å^2^ < *X* ≤ 90 Å^2^, which ensures the best efficacy to permeate the blood–brain barrier, whereas out of this range is considered undesirable [62]. Only the control ligand can cross the blood–brain barrier among the five ligands since the PSA value of serpentine was to be found within the ideal range (Fig. 7H).

Although the research gained significant results in all aspects of “*in silico* molecular drug designing” for receptor specificity, few limitations also have been experienced. Firstly, certification of the ligands used in this study as targeted therapeutics cannot be carried out as long as no *in vivo* and clinical trial occurs. Secondly, the ligands of interest may show some mild to moderate side effects among the experimental subjects (animals like mice, rats, etc.). For instance, overuse of apigenin can cause stomach discomfort [63]; isovitexin can cause anxiety, affecting the brain cells [64]; serpentine shows hepatotoxicity sometimes [http://biosig.unimelb.edu.au/pkcsm/]; irritation and burning of the eyes, nose, throat, and skin in humans can emerge for piperolactam A; nausea, headache, and shortness of breath may sometimes result from quercetin overuse [https://www.rxlist.com/consumer_quercetin/drugs-condition.htm]. Proper consideration of the effects of the dose is essential for these kinds of ligands. 

## Conclusion

This *in silico* study was conducted through a series of methodical procedures, which included: establishment of a phytochemical repository of 120 ligands based on a comprehensive literature review; selection of five top ligands including control based on drug likeliness through ADMET and QSAR-based ligand screening; in-depth analysis of the level of interaction among the receptor protein ACE2 (1R4L) and the respective test ligands through molecular docking of these ligands against the target receptor, optimized to a high-resolution crystalized level; and finally molecular dynamic simulation to attain the quantitative values for RMSD, RMSF, α-carbon, intra hydrogen bond, radius of gyration, SASA, MolSA, and PSA to observe the extent of the mobility of protein induced when in association with prospective drugs. Molecular docking analysis found all the test ligands to exhibit substantial binding affinity with the targeted macromolecule, while the Ligplot+ visualization demonstrated potential hydrogen and non-covalent bonds responsible for the strength of protein–ligand affinity. Moreover, the MDS suggested the hierarchy of five test ligands concerning least mobility of protein backbone, least fluctuation of protein alpha carbons, least flexibility of protein in complex with ligand, least available atomic van der Waals surface area, and protein surface area exposed to water for the protein–ligand complex, with a surface associated with heteroatoms and hydrogen atoms under desirable range. This study prognosticated apigenin, piperolactam A, and quercetin to be more stable and highly interactive flavonoid compounds. Thus, further *in vivo* investigation is recommended to determine their therapeutic potentiality and target specificity toward the target ACE2 receptor so that the SARS-CoV-2 influx can be prevented and the virus-derived heart injury can be protected. Although isovitexin exhibited the highest binding tendency with the hACE2 receptor, it was not suggested as the superior drug-like compound due to its low pharmacodynamics stability observed in MDS than the others. 

## Acknowledgments

The authors are grateful to the RPG (Govt. License ID: 05-060-06021) for providing all types of unconditional supports in the technical issues (under the Project of Category: F4; ID. #09-2021/22). The authors are also grateful to Dr. Sharmin Ahmed (Chulalongkorn University, Thailand) and Mr. Parag Kumar Paul (Dept. of Electrical and Electronic Engineering, United International University, Bangladesh) for their support in final plagiarism and grammatical checking.

## List of abbreviations

SARS-CoV-2: Severe acute respiratory syndrome coronavirus 2; COVID-19: Coronavirus disease 2019; S protein: Spike protein; hACE2: Human angiotensin-converting enzyme 2; ADMET: Absorption, distribution, metabolism, and excretion; QSAR: Quantitative structure–activity relationship; PSA: Polar surface area; RMSD: Root mean square deviation; RMSF: Root mean square fluctuation; PDB: Protein data bank; MDS: Molecular dynamics simulations; TC: Total clearance; LARMD: Ligand and receptor molecular dynamics; Rg: Radius of gyration; Cα: Alpha carbon; SASA: Solvent accessible surface area; MolSA: Molecular surface area; H-bonds: Intramolecular hydrogen bonds 
